# Paving the way for predictive diagnostics and personalized treatment of invasive aspergillosis

**DOI:** 10.3389/fmicb.2015.00411

**Published:** 2015-05-05

**Authors:** Ana Oliveira-Coelho, Fernando Rodrigues, António Campos, João F. Lacerda, Agostinho Carvalho, Cristina Cunha

**Affiliations:** ^1^Life and Health Sciences Research Institute (ICVS), School of Health Sciences, University of Minho, Braga, Portugal; ^2^ICVS/3B’s - PT Government Associate Laboratory, Braga/Guimarães, Portugal; ^3^Serviço de Transplantação de Medula Óssea, Instituto Português de Oncologia do Porto, Porto, Portugal; ^4^Instituto de Medicina Molecular, Faculdade de Medicina de Lisboa, Lisboa, Portugal; ^5^Serviço de Hematologia e Transplantação de Medula, Hospital de Santa Maria, Lisboa, Portugal

**Keywords:** invasive aspergillosis, stem cell transplantation, antifungal immunity, single nucleotide polymorphism, personalized medicine

## Abstract

Invasive aspergillosis (IA) is a life-threatening fungal disease commonly diagnosed among individuals with immunological deficits, namely hematological patients undergoing chemotherapy or allogeneic hematopoietic stem cell transplantation. Vaccines are not available, and despite the improved diagnosis and antifungal therapy, the treatment of IA is associated with a poor outcome. Importantly, the risk of infection and its clinical outcome vary significantly even among patients with similar predisposing clinical factors and microbiological exposure. Recent insights into antifungal immunity have further highlighted the complexity of host-fungus interactions and the multiple pathogen-sensing systems activated to control infection. How to decode this information into clinical practice remains however, a challenging issue in medical mycology. Here, we address recent advances in our understanding of the host-fungus interaction and discuss the application of this knowledge in potential strategies with the aim of moving toward personalized diagnostics and treatment (theranostics) in immunocompromised patients. Ultimately, the integration of individual traits into a clinically applicable process to predict the risk and progression of disease, and the efficacy of antifungal prophylaxis and therapy, holds the promise of a pioneering innovation benefiting patients at risk of IA.

## Introduction

Aspergillosis includes an extensive spectrum of diseases caused by fungi of the genus *Aspergillus* with clinical manifestations that range from colonization to allergic bronchopulmonary aspergillosis and disseminated forms of infection (Segal, 2009). The prevalence of invasive aspergillosis (IA) has steadily increased in the last decades, mostly due to the advent of solid organ and hematopoietic stem cell transplantation (HSCT), and the increased use of chemotherapy and immunosuppression (Kontoyiannis et al., 2010; Pagano et al., 2010). Although the diagnosis of IA has improved, namely because of the introduction of biomarkers such as the detection of galactomannan in the clinical practice (Morrissey et al., 2013), successful treatment is still a challenging endeavor. Indeed, established infection is difficult to eradicate, resulting in associated mortality rates ranging from 40 to 90% (Walsh et al., 2008).

The extensive use of empiric antifungal therapy is a major concern given the potential development of resistance in the pathogen. The emergence of azole-resistant strains of *Aspergillus fumigatus* (Steinmann et al., 2015) underscores the need for personalized diagnostic and risk prediction approaches based on individual traits that may allow targeting antifungal therapy and improving outcomes without unnecessary empiric therapy. Several host genetic variants have been proposed as suitable markers to categorize patients with the highest risk of infection (Cunha et al., 2013). However, many challenges confront the development of these predictive tools. Among them, our insufficient understanding of the critical immune defects that predispose to the infection and the lack of validation of genetic diagnostics in well-designed clinical trials.

The purpose of this review is to reposition novel findings on the host-fungus interaction and to discuss the challenges in exploiting this knowledge to the design of patient-tailored diagnostic or therapeutic approaches to deliver to specific target populations.

## The Host-fungus Interaction: What’s New?

The physical barriers afforded by the respiratory mucosa confer the first line of innate resistance to fungal infection. Because of their small size, conidia may reach the lung alveoli, where they are phagocytosed and killed through the production of reactive oxygen species (ROS) by resident macrophages, whereas neutrophils are instead proficient at handling hyphae germinating from conidia that escape macrophage surveillance through an array of extracellular killing mechanisms (Ibrahim-Granet et al., 2003; Cunha et al., 2014b; Heinekamp et al., 2015). This morphotype preference may be in part due to the ability of neutrophils to sense microbe size and selectively release neutrophil extracellular traps in response to larger structures (Branzk et al., 2014). Importantly, recent findings also suggest that lung epithelial cells act as an active extension of the innate immune system, operating as a surveillance mechanism sensing fungal spores and prompting antifungal effector responses (Osherov, 2012).

Innate immune cells are equipped with pattern recognition receptors (PRRs) able to discriminate pathogen-associated molecular patterns (PAMPs; Bourgeois and Kuchler, 2012; Plato et al., 2015). Stimulation of antigen-presenting cells, including macrophages and dendritic cells (DCs), leads to the activation and recruitment of lymphocytes, and the development of adaptive antifungal immune responses. Once committed, T cells monitor the host for infection and mobilize appropriate effector functions by inducing cytokines and cytolytic molecules, which are instrumental in rallying and activating professional phagocytes to the site of microbial deposition, thus providing a prompt and effective control of infection (Romani, 2011).

The fungal cell wall is the main source of PAMPs owing to its dynamic composition and structural properties according to morphotype, growth stage and environmental conditions (Latge, 2010). Toll-like receptor (TLR)-2 (in cooperation with TLR1 and TLR6), TLR3, TLR4, and TLR9, and the C-type lectin receptors dectin-1, dendritic cell-specific intercellular adhesion molecule 3 grabbing non-integrin (DC-SIGN) and mannose receptor are the most important PRRs recognizing fungal PAMPs including mannan, β-glucan and nucleic acids (Romani, 2011).

### The Host Perspective

The immune response to *A. fumigatus* is determined not only by the relative degree of stimulation of the individual PRRs but also by the level of receptor cooperation and cellular localization. Indeed, a sequential activation of distinct signal transduction pathways through the PRR adapters myeloid differentiation primary response 88 (MyD88) and caspase-associated recruitment domain 9 (CARD9) in the respiratory epithelium and hematopoietic compartment in response to infection was recently reported (Jhingran et al., 2015). By means of a partial overlap, these signals ensure optimal chemokine induction, neutrophil recruitment, and fungal clearance within the respiratory tract.

Patients undergoing immunosuppressive regimens based on calcineurin inhibitors such as cyclosporine A or corticosteroids are highly susceptible to disseminated fungal infections. Previous work demonstrated that calcineurin is an important regulator of dectin-1-mediated signaling and activation of immunity to *Candida albicans* (Greenblatt et al., 2010), and that corticosteroid immunosuppression blocks dectin-1-mediated signaling required for maturation of *A. fumigatus* phagosomes (Kyrmizi et al., 2013). More recently, the calcineurin inhibitor tacrolimus was found to impair primary alveolar macrophage activation in response to *A. fumigatus* by preventing a signaling pathway involving a TLR9-Bruton’s tyrosine kinase-calcineurin-nuclear factor of activated T-cells axis required for proinflammatory cytokine production (Herbst et al., 2015). Taken together, these findings suggest that disseminated fungal infections seen in these patients are not just a general consequence of systemic suppression of adaptive immunity but are, rather, a result of the specific blockade of evolutionarily conserved innate pathways for fungal resistance.

Another important feature of dectin-1-mediated signaling in response to *A. fumigatus* is the activation of the nucleotide-binding oligomerization domain-like receptor family, pyrin domain containing 3 (NLRP3) inflammasome leading to the production of bioactive interleukin (IL)-1β (Said-Sadier et al., 2010). Indeed, members of the IL-1 receptor family of cytokines are critical effector molecules in antifungal immunity (Gresnigt and van de Veerdonk, 2014). By preventing activation of the NLRP3 inflammasome and reducing IL-1β secretion, IL-37 was recently found to act as a broad spectrum inhibitor of innate responses to fungal infection-mediated inflammation (Moretti et al., 2014). Along this line, the increased IL-1β release intrinsically associated to chronic granulomatous disease (CGD) was found to be reverted by the use of IL-1 receptor antagonist (IL-1Ra), leading to restrained neutrophil recruitment and T helper 17 responses, thereby protecting from IA (de Luca et al., 2014).

Soluble PRRs found in the fluids lining the epithelial surfaces support fungal sensing by binding to conidia and enhancing their uptake by phagocytes. Among these, the long pentraxin 3 (PTX3) was demonstrated to play a non-redundant role in antifungal host defense (Garlanda et al., 2002) by enhancing recognition and phagocytosis through mechanisms that depend on Fcγ receptor, CD11b and complement activation (Moalli et al., 2010). Engagement of myeloid differentiation protein 2 (MD-2) during uptake of PTX3-opsonized conidia was also revealed to activate TLR4 signaling converging on the production of type I interferons (Bozza et al., 2014). This suggests that in addition to pro-phagocytic properties, PTX3 is able to elicit antifungal effector mechanisms associated with limited immunopathology, thereby highlighting potential mechanisms of action underlying its favorable synergism with antifungal therapy against IA (Lo Giudice et al., 2012; Marra et al., 2014).

### The Pathogen Perspective

Fungi have evolved their own elaborate mechanisms to escape innate immunity. By masking dectin-1 and dectin-2-dependent recognition, the hydrophobin layer of *A. fumigatus* conidia restrains neutrophil infiltration and cytokine production (Aimanianda et al., 2009; Carrion Sde et al., 2013). Galactosaminogalactan (GAG), a polysaccharide of the fungal cell wall produced by glucose epimerases (Lee et al., 2014), also hampers neutrophil recruitment (Fontaine et al., 2011). GAG functions as an adhesin, mediating adherence and suppressing host inflammatory responses, in part through masking cell wall β-glucan from recognition by dectin-1 (Gravelat et al., 2013). The immunosuppressive properties of GAG have also been attributed to its potent ability to induce IL-1Ra (Gresnigt et al., 2014), a finding further highlighting a possible therapeutic option targeting IL-1Ra in IA.

Genomic and transcriptomic approaches have revealed that fungal pathogenicity depends also on mechanisms regulating fungal metabolism and response to stress in adaptation to the host environment. In particular, the ability of *A. fumigatus* to adapt to hypoxic microenvironments has been found to involve the production of secondary metabolites that promote lung inflammation, exacerbate infection and influence subsequent host immune responses (Grahl et al., 2011). Given the need of myeloid cells to adapt to hypoxic and inflamed microenvironments that develop during infection, the hypoxia-inducible factor 1-alpha (HIF-1α) has been found to be essentially required for chemokine production and maintenance of neutrophil numbers in the lungs of infected animals (Shepardson et al., 2014).

Effector T cell responses are also targeted by fungi. For example, mucosal vaccination was found to subvert T cell priming by impairing chemokine signals on egress of inflammatory monocytes from the bone marrow and their recruitment to the lung (Wuthrich et al., 2012). This finding is even more significant considering the role of inflammatory monocytes in orchestrating antifungal immunity in the lung by regulating the conidiocidal activity of neutrophils and their own differentiation to DCs (Espinosa et al., 2014). Importantly, inflammatory monocytes are required for optimal IL-1α expression in the lungs, which in turn regulates the early accumulation of neutrophils in the lung (Caffrey et al., 2015).

## Host Genetic Determinants of Risk of IA

The inborn deficiency of the phagocyte nicotinamide adenine dinucleotide phosphate (NADPH) oxidase leading to a defective production of ROS and underlying CGD is the best known example of primary immunodeficiency associated with a distinctive predisposition to IA (Vinh, 2011). Patients with autosomal-dominant hyper-IgE syndrome (AD-HIES) are also at risk for IA (Vinh et al., 2010); however, the susceptibility of AD-HIES patients classically results from concurrent anatomical lung defects from previous bacterial infections and defective STAT3-dependent epithelial immunity (Holland et al., 2007). For most individuals however, the genetic propensity to IA has a polygenic source that acts in combination with other remarkable predisposing variants (e.g., the profound immunosuppression typical of many clinical settings), and that may translate into further immunological dysfunction, ultimately increasing the proneness to infection.

Our increasing ability to analyze human variability at the DNA level has made possible the identification of genetic factors implicated in the development of IA in hematological patients (Cunha et al., 2013), providing important insights into the mechanisms of human diversity underlying increased susceptibility to IA. Although genetic profiling is nowadays regarded as a promising methodology to exploit in the future toward improved diagnosis and therapy of fungal diseases (Cunha and Carvalho, 2012), this field is still bedeviled by difficulties, mostly related to heterogeneity of cohorts, sample size, selection bias, and statistical flaws (van der Velden et al., 2011), which compromise the clinical applicability of this knowledge.

The identification of a donor haplotype in *TLR4* increasing risk of IA after HSCT was one of the first solid reports illustrating the remarkable influence of host genetics on the susceptibility to IA (Bochud et al., 2008). This phenotype was associated with a delayed T cell and natural killer T cell immune reconstitution after transplant (Koldehoff et al., 2013). However, despite *TLR4* polymorphisms have also been linked with chronic aspergillosis in immunocompetent individuals (Carvalho et al., 2008) and fungal colonization in HSCT recipients (Carvalho et al., 2009), their prognostic significance remains unclear, since the exact mechanism(s) through which TLR4 deficiency impacts human antifungal immunity remain to be identified. Along this line, the early finding that genetic variants in *TLR1* and *TLR6* predisposed to IA among HSCT recipients (Kesh et al., 2005) was recently supported by evidence demonstrating the defective production of crucial antifungal cytokines and chemokines by TLR1- and TLR6-deficient mouse cells after stimulation with *A. fumigatus* (Rubino et al., 2012).

The discovery that sensing of fungal RNA by TLR3 was required for the activation of protective memory CD8^+^ T cells responses in experimental aspergillosis was complemented by the identification of a regulatory variant impairing the expression of the human receptor in human DCs and hampering the efficient priming of memory CD8^+^ responses (Carvalho et al., 2012b). These findings suggest that, by interpreting immunogenetic signatures and identifying subtle differences in immune profiles, response efficiencies to potential antifungal vaccination strategies are likely to be discriminated. In fact, the measurement of *A. fumigatus*-specific immune responses in hematological patients was confirmed as a promising immunodiagnostic approach (Potenza et al., 2013) amenable to combination with other diagnostic tools. Finally, a stop codon in recipient TLR5 was also disclosed as an important prognostic factor for the development of IA among HSCT recipients (Grube et al., 2013), a finding warranting further studies into the function of this receptor in antifungal immunity.

Dectin-1 deficiency was also consistently reported to contribute to susceptibility to IA (Cunha et al., 2010; Chai et al., 2011; Sainz et al., 2012). The finding that by compromising the surface expression and dectin-1-mediated cytokine production, the presence of the Y238X polymorphism in HSCT donors and recipients displayed a cumulative effect toward risk for infection (Cunha et al., 2010) emphasizes the contribution of non-hematopoietic dectin-1 to antifungal immunity. In addition, although damage perception is fundamental for resolution of fungal infection (Cunha et al., 2012), genetic variants triggering hyperactive danger signaling, and presumably leading to uncontrolled inflammatory responses to the fungus, were also recently found to increase risk for IA (Cunha et al., 2011).

A number of positive associations between genetic variants in cytokine and chemokine genes and vulnerability to IA has also been reported (Sainz et al., 2007a,b, 2008, 2010; Mezger et al., 2008; Carvalho et al., 2010). One recent example regards the identification of polymorphisms in the genes encoding for IL-1β and beta-defensin 1 (*DEFB1*) that, by affecting production of *A. fumigatus*-induced proinflammatory cytokines by mononuclear cells, influenced susceptibility to mold infection after solid organ transplantation (Wojtowicz et al., 2014).

A number of unconventional strategies have been employed to uncover additional candidate genes for susceptibility to IA (Zaas et al., 2008; Durrant et al., 2011). For example, genetic mapping analysis of survival data of infected mice allowed the identification of plasminogen, a regulatory molecule with opsonic properties, as a fitting contestant for susceptibility (Zaas et al., 2008). Consequently, a non-synonymous polymorphism in human plasminogen was found to increase risk for IA in HSCT recipients. Genetic and functional deficiency of other molecules with opsonic activity—e.g., mannose-binding lectin (MBL; Lambourne et al., 2009) and PTX3 (Cunha et al., 2014a)—has also been disclosed as a major determinant of susceptibility to IA, pointing to a key contribution of innate humoral responses to antifungal immunity. Indeed, a donor haplotype in *PTX3* associated with increased risk of IA in the corresponding HSCT recipient was found to compromise PTX3 expression during the developmental programming of neutrophils in the bone marrow, leading to a defective antifungal capacity of newly reconstituted neutrophils (Cunha et al., 2014a). Importantly, this association was recently replicated in a cohort of solid organ transplant recipients (Wojtowicz and Bochud, 2015). The fact that exogenous administration of PTX3 is able to revert the genetic defect (Cunha et al., 2014a) further highlights the potential of PTX3-based immunotherapies to treat (or prevent) IA (Carvalho et al., 2012a).

## Decoding the Host-fungus Interaction into Clinical Strategies

Early diagnosis is crucial to a favorable outcome of IA. However, the existing diagnostic tools are often compromised by slowness, invasiveness, lack of standardization and insufficient understating of their kinetics (Hope et al., 2005). The introduction of molecular and serological diagnostic techniques into clinical practice has undoubtedly improved our capacity to diagnose IA. Nonetheless, the broad applicability of both techniques is hampered by considerable variability in performance. Given these technical barriers, the search for tools to diagnose IA that are more efficient and reliable is an active field of research. One example was recently provided by a study demonstrating the usefulness of direct detection of exogenous fungal metabolites in the breath to the identification of the underlying microbial etiology of pneumonia (Koo et al., 2014).

**FIGURE 1:**
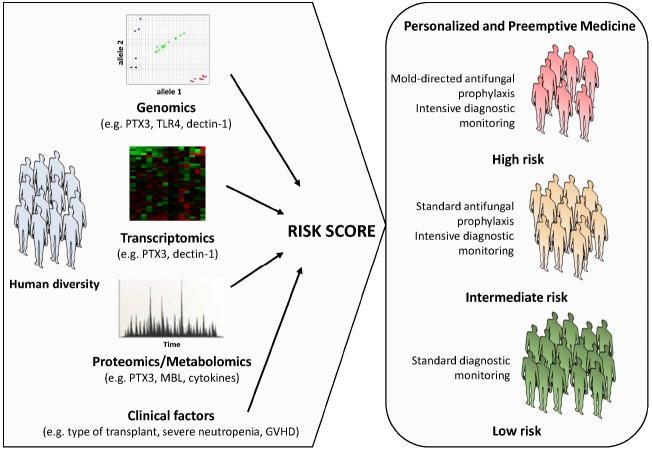
**Schematic representation of a personalized medicine approach to the management of IA.** A prognostic score calculated using information on host biomarkers and clinical factors is used to determine the risk of IA associated to a given patient profile. The individual aspects of the patient (and donor), including the genetic make-up and downstream activated transcriptomic and proteomic or metabolomic networks, as well as inherent clinical factors are directly considered to guide treatment planning. Although the risk category is defined at initiation of treatment (or before stem cell transplantation), it may be updated in the course of treatment according to the clinical status of the patient (e.g., development of graft-versus-host-disease, prolonged neutropenia, etc.). A number of host genetic variants in innate immunity genes (e.g., PTX3, TLR4, dectin-1, MBL, and several cytokines) have been disclosed as promising targets to use in patient-tailored strategies to optimize and target the diagnostic workup, and the antifungal prophylaxis and therapy, thereby improving patient outcome.

Although the interaction of the fungus with the immune system is being exploited to project novel and improved fungal diagnostics, efforts have on the other hand been also devoted to the implementation of clinical models aimed at the prediction of infection in high-risk patients. In this regard, interpretation of individual genomic, transcriptomic, proteomic or metabolomic profiles associated with impaired antifungal immune responses and their integration with clinical data is regarded as a promising approach (Figure 1). Indeed, next-generation sequencing technologies now provide exciting possibilities to pin down essential steps in host-fungus interaction at a level of complexity previously unanticipated. The first genome-wide association studies (GWAS) exploring host susceptibility to IA are underway and are expected to provide unbiased insights into the genetic defects contributing to development of IA, thereby laying the foundation for clinical trials aimed at the validation of medical interventions based on individual genomics. These efforts are nonetheless centered on the fairly “static” role played by the genetic variants. Physiological responses to fungal infection require the coordinated regulation of gene expression, which may vary markedly between individuals and influence phenotypes such as protein levels, the cell morphology and function, and ultimately the immunity to infection. Thus, genetic analysis of molecular traits such as the gene expression represents a powerful approach enabling insights into the human genomic landscape by generating expression maps useful for the functional interpretation of non-coding variants likely to arise from ongoing genome-wide initiatives (Fairfax and Knight, 2014).

## Conclusions and Perspectives

The discovery of accurate and reliable genetic markers of susceptibility may be a turning point toward innovative stratification strategies based on genetic screening or immune profiling to predict risk and severity of disease, efficacy of antifungal prophylaxis and therapy, and eventually contribute to the successful design of antifungal vaccines. As shown for PTX3 deficiency (Cunha et al., 2014a), targeting cell function (e.g., exogenous administration of lacking or deficient factors) may prove an interesting approach to be validated in the future. Indeed, engineering of T cell function to target carbohydrates was demonstrated as a potentially exploitable strategy for the treatment of IA (Kumaresan et al., 2014). Ultimately, approaches based on individual genomics (and with influence on multiple functional transcriptomic, proteomic and metabolomic networks) may warrant important clinical tools allowing discrimination of patients that require enhanced surveillance for fungal disease or alternative antifungal therapies.

### Conflict of Interest Statement

The authors declare that the research was conducted in the absence of any commercial or financial relationships that could be construed as a potential conflict of interest.
